# Variation of LDL cholesterol in response to the replacement of saturated with unsaturated fatty acids: a nonrandomized, sequential dietary intervention; the Reading, Imperial, Surrey, Saturated fat Cholesterol Intervention (“RISSCI”-1) study

**DOI:** 10.1016/j.ajcnut.2024.07.032

**Published:** 2024-08-05

**Authors:** Athanasios Koutsos, Bruce A Griffin, Rona Antoni, Ezgi Ozen, Laury Sellem, Gloria Wong, Hasnaa Ayyad, Barbara A Fielding, MD Robertson, Jonathan Swann, Kim G Jackson, Julie A Lovegrove

**Affiliations:** 1 Hugh Sinclair Unit of Human Nutrition, Department of Food and Nutritional Sciences, Institute for Food, Nutrition and Health, and Institute for Cardiovascular and Metabolic Research, University of Reading, Reading, United Kingdom; 2 Department of Nutrition, Food and Exercise Sciences, Faculty of Health & Medical Sciences, University of Surrey, Guildford, United Kingdom; 3 School of Human Development and Health, Faculty of Medicine, University of Southampton, Southampton, United Kingdom

**Keywords:** cardiovascular disease, dietary fat replacement, saturated and unsaturated fatty acids, LDL cholesterol, gene expression, *APOE* genotype, interindividual variation, nuclear magnetic resonance, lipoprotein subfractions, E-selectin

## Abstract

**Background:**

Serum low density lipoprotein (LDL) cholesterol shows marked interindividual variation in response to the replacement of saturated fatty acids (SFAs) with unsaturated fatty acids (UFAs).

**Objectives:**

To demonstrate the efficacy of United Kingdom guidelines for exchanging dietary SFAs for UFAs, to reduce serum LDL cholesterol and other cardiovascular disease (CVD) risk factors, and to identify determinants of the variability in LDL cholesterol response.

**Methods:**

Healthy males (*n* = 109, mean ± SD age 48 ± 11 y; BMI 25.1 ± 3.3 kg/m^2^), consumed a higher-SFA/lower-UFA diet for 4 wk, followed by an isoenergetic, lower-SFA/higher-UFA diet for 4 wk (achieved intakes SFA:UFA as % total energy 19.1:14.8 and 8.9:24.5, respectively). Serum LDL cholesterol, CVD risk markers, peripheral blood mononuclear cell (PBMC) gene expression, and dietary intakes were assessed at baseline and the end of each diet.

**Results:**

Transition from a higher-SFA/lower-UFA to a lower-SFA/higher-UFA diet significantly reduced fasting blood lipids: LDL cholesterol (−0.50 mmol/L; 95% confidence interval [CI]: −0.58, −0.42), high-density lipoprotein (HDL) cholesterol (−0.11 mmol/L; 95% CI: −0.14, −0.08), and total cholesterol (TC) (−0.65 mmol/L; 95% CI:−0.75, −0.55). The dietary exchange also reduced apolipoprotein (apo)B, TC:HDL cholesterol ratio, non-HDL cholesterol, E-selectin (*P* < 0.0001), and LDL subfraction composition (cholesterol [LDL-I and LDL-II], apoB100 [LDL-I and LDL-II], and TAG [LDL-II]) (*P* < 0.01). There was also an increase in plasma biomarkers of cholesterol intestinal absorption (β-sitosterol, campesterol, cholestanol), and synthesis (desmosterol) (*P* < 0.0001) and fold change in PBMC *LDL-receptor* mRNA expression relative to the higher-SFA/lower-UFA diet (*P* = 0.035). Marked interindividual variation in the change in serum LDL cholesterol response (−1.39 to +0.77 mmol/L) to this dietary exchange was observed, with 33.7% of this variation explained by serum LDL cholesterol before the lower-SFA/higher-UFA diet and reduction in dietary SFA intake (adjusted *R*^2^ 27% and 6.7%, respectively). *APOE* genotype was unrelated to serum LDL cholesterol response to SFA.

**Conclusions:**

These findings support the efficacy of United Kingdom SFA dietary guidelines for the overall lowering of serum LDL cholesterol but showed marked variation in LDL cholesterol response. Further identification of the determinants of this variation will facilitate targeting and increasing the efficacy of these guidelines.

The RISSCI-1 study was registered with ClinicalTrials.Gov (No. NCT03270527).

## Introduction

There is strong and consistent evidence supporting a causal role of elevated serum LDL cholesterol in the development of atherosclerotic cardiovascular disease (CVD) [1,2], and a marked reduction in LDL cholesterol when replacing dietary saturated fatty acids (SFAs) with unsaturated fatty acids (UFAs) [3,4]. These 2 phenomena underlie the recommendation to reduce dietary SFA, which has stood as a cornerstone of guidelines for the prevention of CVD for over 60 y [3,5]. However, interindividual variation in serum LDL cholesterol in response to this dietary exchange, in the order of 0.5–1 mmol/L, has been observed in intervention trials [6], the National Cholesterol Education Program low SFA Step 2 diet [7], and retrospectively in our own studies with similar interventions [8]. Interindividual variation in LDL cholesterol response has also been reported in the absence of changes in dietary polyunsaturated fatty acids in males with obesity on a very high fat, low carbohydrate diet [9]. The response of serum LDL cholesterol to dietary fatty acids is influenced by many factors, including the nature of replacement macronutrients, the nutrient composition and matrix of SFA-rich foods, the chain length of specific SFAs, as well as innate biological differences between individuals [8]. Although a proportion of the variable response in serum LDL cholesterol could also be ascribed to disparities in dietary compliance, the rigorous control of dietary intake in the aforementioned studies implicates differences in genetic and related metabolic traits between individuals. An example of a common genetic trait, that has been reported to render serum LDL cholesterol more or less responsive to dietary SFA, is the *APOLIPOPROTEIN (APO)E* missense polymorphism [10]. This genotype has been shown to contribute to interindividual variation in serum LDL cholesterol response via differential effects on the regulation of the receptor-mediated uptake of serum lipoproteins into cells, and resultant, reciprocal changes in cholesterol biosynthesis and absorption in the intestine [11]. The identification of key biomarkers could increase the efficacy and clinical impact of the dietary guideline to replace SFAs with UFAs in LDL cholesterol responsive individuals. Conversely, it could highlight the need for more intensive intervention or alternative therapeutic approaches in those less responsive to this dietary exchange.

The Reading, Imperial, Surrey, Saturated fat Cholesterol Intervention-1 (“RISSCI”-1) study, aimed to determine the impact of current guidelines to reduce and replace SFA with UFA on traditional CVD risk factors (including anthropometric measures, fasted lipid profile, blood pressure, glucose, and insulin), and more novel markers (inflammatory markers and cell adhesion molecules) to identify and characterize the distribution of interindividual variation in the response of serum LDL cholesterol. Possible determinants of this variability included lipid-related gene expression in peripheral blood mononuclear cells (PBMCs), markers of cholesterol synthesis and intestinal absorption, and lipoprotein subfractions measured by nuclear magnetic resonance (NMR).

## Methods

### Study design and participants

The study was designed as a nonrandomized, 2 sequential, 4-wk, single-blind dietary intervention; a higher-SFA/lower-UFA “run-in” diet, followed by a lower-SFA/higher-UFA diet, with no washout period. This study design was chosen specifically to reproduce the effects of the transition from a relatively high SFA intake (18% of total energy [TE]) to a lower intake, to align with the United Kingdom dietary guideline (10% of TE from SFA) [3].

The dietary interventions were conducted at the Universities of Reading and Surrey, with recruitment taking place from September 2017 to June 2019 and the interventions concluding in September 2019. A participant flow chart can be found in the Online Supplementary Material, Supplementary Figure 1. Healthy males, aged 30−65 y with BMI of 19−32 kg/m^2^ were recruited using volunteer databases, posters, and social media. After completing a health and lifestyle questionnaire, eligible participants attended a screening visit after a 12-h fast, 2−4 wk before the trial. Participants provided informed verbal and written consent, followed by screening measurements of height, weight, blood pressure, and biochemistry (blood lipids, liver and kidney function tests, and full blood count, performed at the Departments of Pathology at the Royal Berkshire, and Royal Surrey County Hospitals). Participants with results outside clinical reference ranges were excluded. Details of the inclusion and exclusion criteria and clinical reference ranges have been previously published [12]. Briefly, at enrolment, disease-free participants were instructed to maintain their regular physical activity regime, and to promptly report any changes in health status or use of medication to the research teams.

The RISSCI-1 study received favorable ethical opinions for conduct from Research Ethics Committees at the University of Reading (UREC 17/29) and University of Surrey (UEC/2017/41/FHMS). The study was performed in accordance with the Declaration of Helsinki guidelines. Participants provided written informed consent before participating. The RISSCI-1 study was registered with ClinicalTrials.gov (NCT03270527).

### Dietary intervention

Replacement of dietary SFAs with MUFAs/PUFAs (UFAs) was achieved by a food exchange model, which included 2, 4-wk, isoenergetic, moderate-fat diets (38% TE from fat). Details of our dietary intervention have been described previously [12]. Briefly, participants followed: diet 1, higher in SFA/lower in MUFA/PUFA (UFA), %TE SFA:MUFA:PUFA 18:12:4; and diet 2, lower in SFA/higher in MUFA/PUFA (UFA), %TE SFA:MUFA:PUFA 10:14:10, each for 4 wk. Both diets were matched for total energy, dietary fat, and other macronutrients. Compliance was assessed using 4-d weighed diet diaries, completed before each study visit, daily tick sheets, and by measuring plasma phospholipid fatty acids as a short-term biomarker of fatty acid intake [12].

Participants attended 3 study visits after a 12-h fast: Visit 1 (wk 0) at baseline, Visit 2 (wk 4) after completion of the 4-wk higher-SFA/lower-UFA diet, and Visit 3 (wk 8) after the 4-wk lower-SFA/higher-UFA diet. At each visit, anthropometrics (height, weight, waist, and hip circumferences), body fat composition (Tanita BC-418 digital scale), and blood pressure (using a Mobil-O-Graph Ambulatory Blood Pressure Monitor (IEM GmbH)) were measured, and a blood sample was taken.

### Outcomes

The outcomes measured in response to the dietary intervention are described below.

### Blood biochemical analysis

Serum lipids (total cholesterol [TC], HDL cholesterol, triacylglycerol [TAG], and non-esterified fatty acids [NEFAs]), apoB, glucose, and C-reactive protein (CRP) were measured on a Daytona Plus clinical chemistry analyzer, using commercially available kits (Randox Laboratories). Quality controls for inter- and intrabatches were within the reference range specified by the manufacturers. Our primary outcome LDL cholesterol was calculated using the Friedewald formula [13]; non–HDL cholesterol by subtracting the HDL cholesterol from the TC concentration, and remnant lipoprotein cholesterol by subtracting LDL cholesterol from non–HDL cholesterol. Lipoprotein ratios (TC:HDL cholesterol, LDL cholesterol:HDL cholesterol, LDL cholesterol:apoB, and non-HDL:apoB) were calculated as estimates of CVD risk. Serum insulin and protein convertase subtilisin/kexin type 9 (PCSK9) were measured by ELISA kits (Crystal Chem and R&D Systems Europe Ltd., respectively). An adhesion molecule Luminex performance 4-plex assay kit was used for the determination of serum E-selectin, P-selectin, vascular cell adhesion molecule-1 (VCAM-1), and intercellular cell adhesion molecule-1 (ICAM-1) (R&D Systems Europe Ltd.) using a Luminex 200 System (Invitrogen, Thermo Fisher Scientific) with xPONENT software version 4.2.

### NMR lipids and lipoprotein subfractions

The effects of the dietary exchange on plasma lipids and lipoprotein subfractions were examined by ^1^H-NMR spectroscopy. This was performed on a 600 MHz AVANCE III NMR spectrometer (Bruker BioSpin) equipped with a SampleJet autosampler held at 6°C. Plasma samples (300 μL) were combined with 300 μL of sodium phosphate buffer (80% H_2_O, 20% D_2_O) containing the internal standard, trimethylsilypropionate (TSP), and bacteriostatic, sodium azide. Samples were vortexed to mix, centrifuged at 10,000 × *g* for 10 min at 4°C, and transferred to 5 mm NMR tubes. One-dimensional NMR spectra were acquired following the standard operating procedure for the Bruker *in vitro* Diagnostic research platform [14]. From the spectrum of each sample, plasma lipids and lipoprotein subfractions (VLDL, LDL, and HDL) were quantified using Bruker B.I. LISA lipoprotein subclass analysis. This identified 6 LDL, 5 VLDL, and 4 HDL subfractions. The LDL subfractions were subsequently grouped, according to density and particle size, into 3 LDL subfractions, corresponding to LDL-I (large size), II (intermediate size), and III (small size) as resolved by density gradient ultracentrifugation and size exclusion, gradient gel electrophoresis (density and size intervals: LDL-I (NMR LDL subfraction 1, 1.019–1.031 kg/L, 27.5–27.0 nm), LDL-II (NMR LDL subfractions 2–5; 1.031–1.044 kg/L, 27.0-25.5 nm), and LDL-III (NMR LDL subfraction 6; 1.044–1.063 kg/L, 25.5–24.2 nm) [15]. Details on the VLDL and HDL subfractions can be found in the Online Supplementary Material.

### Plasma noncholesterol sterols

Plasma noncholesterol sterols were measured as markers of cholesterol intestinal absorption and endogenous synthesis [16] by GC-MS at Newcastle Laboratories, (Newcastle-upon-Tyne Hospitals, NHS Foundation Trust, United Kingdom), using epicoprostanol-5β-cholestan-3a-ol (EPIC) as an internal standard. Briefly, 50 μL plasma was mixed with the internal standard (1 mM EPIC in methanol), and the sterol esters were hydrolyzed under alkaline conditions. The sterols were then double-extracted into hexane. After evaporation of the organic solvent, trimethylsilyl (TMS) derivatives were formed using N, O-Bistrifluroacetamide + 1% TMCS. Samples (1 μL) were injected into the GC-MS and analyzed by single ion monitoring. Plasma noncholesterol sterols; β-sitosterol, cholestanol, and campesterol as markers of cholesterol intestinal absorption; and lathosterol and desmosterol as markers of endogenous cholesterol synthesis, were identified according to their mass spectra and retention time, and quantified by means of standard curves and reference to the concentration of internal EPIC standard. All data were expressed as the ratio of noncholesterol sterol to TC, as measured by GC-MS, to allow for the transport of sterols in plasma lipoproteins [17].

### Gene expression in PBMCs

Blood was collected into a BD Vacutainer cell preparation tube (BD Biosciences) and PBMCs were isolated according to the manufacturer’s instructions. The cell pellet was lysed by the addition of RLT buffer (Qiagen) containing 1% mercaptoethanol before storage at −80°C. Total RNA was isolated using an RNeasy mini kit (Qiagen) according to the manufacturer’s instructions after the cell lysate was passed through a shredder column. RNA quality and quantity were assessed with a Nanodrop ND-1000 spectrophotometer (Thermo Fisher Scientific). cDNA samples were then synthesized from 1.2 μg total RNA using SuperScript IV VILO Mastermix (Thermo Fisher Scientific) and incubated at 25°C for 10 min (reaction volume = 20 μL) followed by 50°C for 10 min and 85°C for 5 min. Samples were diluted 1:10 with UltraPure RNAse/DNAse free distilled water (Invitrogen) and stored at −20°C until further analysis.

Before gene expression analysis, 12 housekeeping genes were screened in a representative subset of cDNA samples from the RISSCI-1 study, using a human geNorm reference gene selection kit (Primerdesign Ltd) and qbase+ software (housekeeping genes: 18S ribosomal RNA, beta-2-microglobulin, beta-actin, eukaryotic initiation factor 4A, glyceraldehyde-3-phosphate dehydrogenase, ATP synthase subunit β (*ATP5B*), DNA topoisomerase I, succinate dehydrogenase complex flavoprotein subunit A, cytochrome c1 (*CYC1*), tyrosine 3-monooxygenase/tryptophan 5-monooxygenase activation protein zeta, ubiquitin C, and ribosomal protein L13a). Expression of the reference and target genes was determined using 5 ng/μL of cDNA by using real-time RT-PCR (QuantStudio3, Life Technologies Limited) with TaqMan gene expression assays (Applied Biosystems) and normal cycling parameters. Expression of each target gene (*LDL-receptor*, sterol regulatory element binding transcription factor 1 [*SREBF1*], nuclear receptor subfamily 1 group H member 3 [*NR1H3*] and ATP-binding cassette subfamily G member 1 [*ABCG1*]) was normalized to the reference genes *CYC1* and *ATP5B* (most stable housekeeping genes). The fold change in mRNA expression relative to the baseline visit for each diet was calculated by the ΔΔCt method expressed as 2^−ΔΔCt^ [18]. Briefly, the C_t_ values of each target gene were normalized to the C_t_ value of the average of the 2 reference genes (ΔCt = C_t target_− C_t reference_), and the relative change calculated to the baseline visit for each diet (ΔΔCt = ΔCt _end of diet_ − ΔCt_baseline visit for the diet_).

### DNA extraction and genotyping

DNA was extracted (Visit 1) from the buffy coat (isolated from blood collected into an EDTA tube), using a DNA blood mini kit (Qiagen Ltd.). DNA samples were retrospectively genotyped for the single nucleotide polymorphisms (SNP) rs429358 and rs7412 to determine the *APOE* genotype (*E2/E4*, *E2/E3*, *E2/E2*, *E3/E3*, *E3/E4*, *or E4/E4*) with the use of TaqMan SNP genotyping assays on the QuantStudio 3 RT-PCR machine.

### Statistical analysis

As this is a proof-of-concept study rather than a confirmatory trial, we chose to adopt a per-protocol analysis approach a priori [19], including data from participants who completed all 3 study visits of the intervention. This approach was taken, rather than an intention to treat, as the study was designed to evaluate efficacy rather than effectiveness [20].

The study was powered on our primary outcome, LDL cholesterol, requiring a total of 92 eligible participants to achieve a 0.16 mmol/L difference in serum LDL cholesterol (SD 0.54) between the higher-SFA/lower-UFA and lower-SFA/higher-UFA diets, at 80% power and 5% level of significance, as previously described [12]. Since TC is composed of LDL cholesterol and HDL cholesterol, the former and latter were also considered primary outcomes. The statistical significance for all primary outcomes was set at *P* < 0.05. Other outcomes were considered secondary and these included anthropometrics (weight, BMI, waist and hip circumferences, and body fat percentage), blood pressure, other blood lipids (TAG and NEFA), lipoprotein ratios (TC:HDL cholesterol, LDL cholesterol:HDL cholesterol, LDL cholesterol:apoB, and non-HDL cholesterol:apoB), non-HDL cholesterol, remnant lipoprotein cholesterol, apoB, NMR lipids and lipoprotein subfractions, glucose, insulin, CRP, and PCSK9; adhesion molecules (serum E-selectin, P-selectin, VCAM-1, ICAM-1), markers of cholesterol intestinal absorption (β-sitosterol, cholestanol, and campesterol), and endogenous cholesterol synthesis (lathosterol, desmosterol). For secondary outcome variables, no formal sample size calculations were performed. *P* < 0.01 was chosen a priori when assessing the significance of these secondary variables to allow for multiple comparisons and identification of interesting findings [21]. Analysis of relative PBMC mRNA gene expression was conducted in a subgroup of participants (*n* = 57 for *SREBF1*, *NR1H3*, *ABCG1*; *n* = 58 for *LDL-R*). Since this analysis was considered exploratory, *P* < 0.05 was chosen as the level of significance, in line with other studies determining PBMC gene expression [22, 23].

Data were analyzed using a linear mixed model with the “*ImerTest*” package in R (version 4.1.2). The model included age (y), BMI (kg/m^2^), baseline measurements (for each response variable), diets (higher-SFA/lower-UFA, lower-SFA/higher-UFA), and study center as fixed effects, and participants as a random effect. Estimated marginal means (EMMs), adjusted for all fixed factors, were presented along with their 95% confidence intervals (CIs). Pairwise comparisons were performed to compare EMMs between the 2 diets using the “*emmeans*” package. For each outcome, participants with missing values resulting from inadequate sample collection or technical errors during analysis were excluded from the statistical analysis. For the PBMC relative mRNA gene expression data, the linear mixed model was adjusted for age and baseline BMI. Data were log transformed if not normally distributed. In such cases, the linear mixed model EMMs on the log scale and pairwise comparisons (Δ, delta) were performed using the “type=response” option in the “emmeans” package, expressing comparisons as ratios of predicted means rather than differences. All EMMs, CIs, and pairwise comparisons are presented on the original untransformed scale to facilitate interpretation.

**FIGURE 1:**
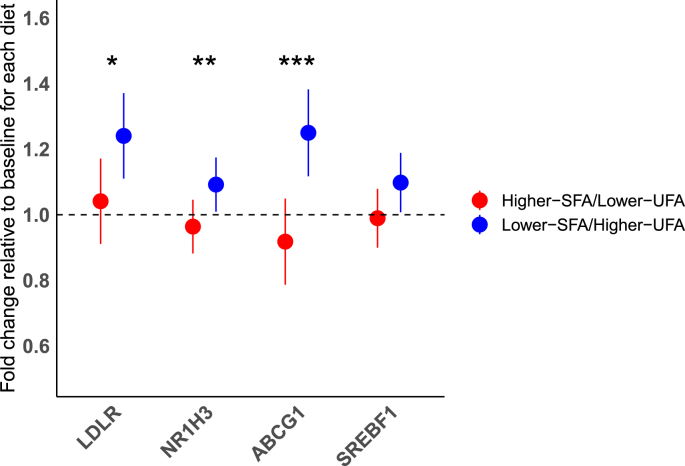
Relative fold change in mRNA gene expression after adult males followed the higher-SFA/lower-UFA and lower-SFA/higher-UFA diets, each for 4 wk. Data are normalized for the reference genes and relative to the baseline visit for each diet, which are arbitrarily set at 1 (represented by the dashed line). Values are estimated marginal means (circles) with 95% confidence intervals (lines) estimated with a mixed linear model adjusted for age and baseline BMI. ^∗^*P* = 0.035, ^∗∗^*P* = 0.009, ^∗∗∗^*P* = 0.0008, *n* = 57 for *SREBF1*, *NR1H3*, *ABCG1*; *n* = 58 for *LDL-R*. *P* = 0.078 for SREBF1. Abbreviations: *ABCG1*, ATP-binding cassette subfamily G member; *LDL-R*, low-density lipoprotein receptor; *NR1H3*, nuclear receptor subfamily 1 group H member 3; *SREBF1*, sterol regulatory element binding transcription factor 1; UFA, unsaturated fatty acids.

In addition to the main analysis, the linear mixed model was also used to assess the differential effects of the 2 diets in relation to the *APOE* genotype carrier code. The potential influence of *APOE* genotype on baseline (visit 1) measurements was evaluated using analysis of variance (ANOVA), with a post hoc Tukey’s honest significance test for multiple comparisons. To present the variability in LDL cholesterol response after replacing dietary SFA with UFA, we performed a waterfall plot showing individual changes in serum LDL cholesterol (Figure 1). Predictive variables affecting this variation in serum LDL cholesterol were examined by stepwise regression (*n* = 52) (in Minitab version 19.2020.2.0), using the difference (delta) in LDL cholesterol (mmol/L) between the higher-SFA/lower-UFA and lower-SFA/higher-UFA diets, as the dependent variable. Outcome measures that showed significant change when SFA was replaced with UFA were selected as potential predictors, along with other variables chosen for their biological relevance to lipid metabolism and cardiovascular health, including anthropometric, dietary, *APOE* genotype, gene expression, and metabolic outcomes. A full list of predictive variables is provided in the Online Supplementary Material. The significance level for variable entry and removal for the stepwise regression was set at a significance level of *P* < 0.05.

## Results

### Baseline characteristics of participants

The baseline characteristics of participants are shown in Table 1. A total of 109 healthy, male participants (mean age 48 (SD 11) y and BMI 25.1 (SD 3.3) kg/m^2^) who completed both dietary fat intervention arms (67 at Reading, 42 at Surrey) were analyzed, from 118 volunteers who enrolled in the study. Participants dropped out because of work commitments (*n* = 5), new medication (*n* = 2), or loss of interest (*n* = 2) **(**Supplementary Figure 1**)**. The participants self-identified their ethnic group as White (86.2%), Asian or Asian British (9.1%), Black, Black British, Caribbean, or African (2.8%), or mixed or multiple ethnic (1.8%) [12]. Carriage of *APOE* alleles were 66% *E3/E3* (*n* = 70), 14% *E2* carriers (*E2/E2* and *E2/E3*, *n* = 15) and 20% E4 carriers (*E4/E4* and *E3/E4*, *n* = 21). At baseline (visit 1) (pre-diets), *APOE4* carriers had significantly higher serum LDL cholesterol, non–HDL cholesterol, and NEFA in comparison to *E2* carriers, with the wild-type *E3/E3* group showing intermediate values **(**Supplementary Tables 1 and 2**).** There were no diet × *APOE* genotype interactions for any of the study outcomes; data are presented in Supplementary Tables 1 and 2).

**TABLE 1:** Anthropometric measurements, blood pressure and CVD risk markers in adult males at baseline and after following the higher-SFA/lower-UFA and lower-SFA/higher-UFA diets, each for 4 wk^1^.

Parameters	*n*	Baseline	Higher-SFA/lower-UFA	Lower-SFA/higher-UFA	Difference (Δ)	*P*
Anthropometrics						
Weight, kg	109	79.6 (77.4, 81.7)	79.5 (79.2, 79.7)	79.3 (79.1, 79.6)	−0.16 (−0.34, 0.01)	0.064
BMI, kg/m^2^	109	25.1 (24.5, 25.7)	25.1 (25.0, 25.2)	25.1 (25, 25.2)	−0.05 (−0.10 0.01)	0.105
Waist circumference, cm	107	92.3 (90.5, 94.1)	91.7 (91.2, 92.2)	91.5 (91.0, 92.0)	−0.17 (−0.65, 0.32)	0.495
Hip circumference, cm	107	102 (101, 103)	102 (101, 102)	102 (101, 102)	−0.08 (−0.52, 0.35)	0.699
Waist:hip ratio	107	0.90 (0.89, 0.91)	0.90 (0.89, 0.90)	0.90 (0.89, 0.90)	0 (−0.01, 0)	0.624
Body fat, %	109	21.4 (20.3, 22.6)	21.3 (21.1, 21.6)	21.1 (20.8, 21.4)	−0.24 (−0.47, −0.01)	0.044
Blood pressure (BP), mmHg						
Systolic BP	100	120 (118, 122)	120 (118, 121)	120 (118, 121)	0.16 (−1.19, 1.51)	0.813
Diastolic BP	102	78 (76.0, 79.1)	77 (75.7, 78.0)	77 (75.6, 78.0)	−0.09 (−1.37, 1.19)	0.893
Pulse pressure	100	43 (40.6, 44.4)	43 (41.6, 44.1)	43 (41.8, 44.4)	0.253 (−1.25, 1.75)	0.739
Blood biochemical analysis						
Total cholesterol, mmol/L	109	5.15 (4.96, 5.34)	5.42 (5.32, 5.53)	4.77 (4.67, 4.88)	−0.65 (−0.75, −0.55)	<0.0001
LDL cholesterol, mmol/L	109	3.18 (3.02, 3.35)	3.38 (3.29, 3.46)	2.88 (2.79, 2.96)	−0.50 (−0.58, −0.42)	<0.0001
HDL cholesterol, mmol/L	109	1.42 (1.36, 1.48)	1.48 (1.45, 1.52)	1.37 (1.34, 1.41)	−0.11 (−0.14, −0.08)	<0.0001
TAG, mmol/L	109	1.21 (1.10, 1.31)	1.24 (1.17, 1.31)	1.15 (1.08, 1.22)	−0.08 (−0.15, −0.01)	0.018
NEFA, mmol/L	108	0.44 (0.40, 0.48)	0.41 (0.38, 0.44)	0.43 (0.40, 0.46)	0.02 (−0.02, 0.06)	0.245
TC:HDL cholesterol ratio	109	3.79 (3.59, 3.99)	3.84 (3.76, 3.93)	3.62 (3.54, 3.71)	−0.22 (−0.29, −0.15)	<0.0001
LDL cholesterol:HDL cholesterol ratio	109	2.37 (2.20, 2.54)	2.42 (2.35, 2.49)	2.21 (2.14, 2.28)	−0.21 (−0.28, −0.15)	<0.0001
Non-HDL cholesterol, mmol/L	109	3.73 (3.54, 3.92)	3.94 (3.84, 4.03)	3.40 (3.31, 3.50)	−0.54 (−0.62, −0.46)	<0.0001
Remnant lipoprotein cholesterol, mmol/L	109	0.55 (0.50, 0.60)	0.56 (0.53, 0.59)	0.52 (0.49, 0.56)	−0.04 (−0.07, −0.01)	0.018
ApoB, g/L	108	0.83 (0.79, 0.87)	0.85 (0.84, 0.87)	0.76 (0.74, 0.78)	−0.09 (−0.11, −0.07)	<0.0001
LDL cholesterol:apoB ratio^3^	108	1.49 (1.46, 1.52)	1.53 (1.50, 1.55)	1.45 (1.43, 1.48)	−0.07 (−0.1, −0.05)	<0.0001
Non-HDL cholesterol:apoB ratio^3^	108	1.74 (1.71, 1.77)	1.78 (1.76, 1.80)	1.72 (1.70, 1.74)	−0.06 (−0.09, −0.04)	<0.0001
Glucose, mmol/L	109	5.24 (5.15, 5.32)	5.22 (5.16, 5.28)	5.24 (5.18, 5.30)	0.03 (−0.04, 0.09)	0.429
Insulin^2^, pmol/L	108	32.8 (23.8, 41.8)	35.6 (33.1, 38.2)	33.6 (31.0, 36.1)	−2.09 (−4.66, 0.49)	0.503
CRP^2^, mg/L	91	1.20 (0.91, 1.49)	1.20 (1.03, 1.37)	1.10 (0.93, 1.27)	−0.10 (−0.26, 0.05)	0.023
VCAM-1^2^, ng/mL	108	474 (443, 505)	467 (448, 486)	475 (456, 494)	7.88 (−11.06, 26.82)	0.364
ICAM-1^2^, ng/mL	108	234 (218, 250)	233 (226, 241)	228 (220, 235)	−5.61 (−13.71, 2.48)	0.262
E-selectin, ng/mL	108	26.4 (24.5, 28.3)	26.5 (25.7, 27.3)	25.1 (24.3, 25.9)	−1.40 (−2.16, −0.64)	<0.0001
P-selectin, ng/mL	108	26.9 (25.5, 28.3)	27.6 (26.8, 28.4)	26.5 (25.7, 27.3)	−1.09 (−1.93, −0.25)	0.011
PCSK9^2^, ng/mL	108	192 (183, 201)	195 (189, 202)	197 (190, 203)	1.08 (−6.02, 8.18)	0.516

Abbreviations: BMI, body mass index; CRP, C-reactive protein; ICAM-1, intercellular cell adhesion molecule 1; *n*, refers to the number of participants for each measured outcome; NEFA, non-esterified fatty acids; PCSK9, protein convertase subtilisin/kexin type 9; TAG, triacylglycerol; TC, total cholesterol; SFA, saturated fatty acid; UFA, unsaturated fatty acid; VCAM-1, vascular cell adhesion molecule 1.

1 Values for higher-SFA/lower-UFA, lower-SFA/higher-UFA and Δ, are estimated marginal means with 95% confidence intervals derived from a mixed linear model adjusted for age, BMI, baseline value of the measured outcome, and study center. Δ (Delta) denotes difference between the 2 diets and is calculated as lower-SFA/higher-UFA minus higher-SFA/lower-UFA. The *P* value represents the pairwise comparisons of the estimated marginal means between the 2 diets. The outcome BMI was adjusted only for age, baseline value, and study center. Baseline (visit 1) indicates values before the dietary intervention and are presented as unadjusted means with 95% confidence intervals. For changes in blood cholesterol (primary outcome) TC, LDL cholesterol, and HDL cholesterol, *P* < 0.05 was considered statistically significant. All other measured outcomes are considered secondary and a more conservative *P* value of <0.01 was considered statistically significant.

2 Indicates data that were log transformed before statistical analysis; these data are presented untransformed and adjusted for age, BMI, baseline value of the measured outcome, and study center.

3 Units of LDL cholesterol, non-HDL cholesterol, and apoB were converted to mg/dL before calculating these ratios.

### Dietary intake and anthropometric measures

The achieved dietary intakes (%TE) were as follows: higher-SFA/lower-UFA diet: SFA:19.1, MUFA:11.1, PUFA:3.7; lower-SFA/higher-UFA diet: SFA:8.9, MUFA:13.4, PUFA:11.1. The intake of total energy, total fat, and other macronutrients were broadly similar between the 2 diets. Further details on dietary intake and measures of compliance have been described previously [12]. There were no significant differences in body weight, BMI, waist and hip circumference, or physical activity level (IPAQ questionnaire, data not shown) between the 2 diets (Table 1).

### Blood biochemical analysis

Isoenergetic replacement of dietary SFA with UFA resulted in significant decreases in serum LDL cholesterol, HDL cholesterol, TC, non-HDL cholesterol, apoB, LDL cholesterol:apoB, non-HDL cholesterol:apoB, TC:HDL cholesterol and LDL cholesterol:HDL cholesterol ratios, and E-selectin (Table 1). Reductions in serum TAG, remnant lipoprotein cholesterol, CRP, and P-selectin were of borderline significance (Table 1).

### NMR lipids and lipoprotein subfractions

Plasma lipids measured by NMR spectroscopy (Table 2) showed broadly similar changes in response to the dietary exchange to the data presented in Table 1. Isoenergetic replacement of dietary SFAs with UFAs resulted in significant reductions in plasma TC and LDL cholesterol. Similarly, there were significant reductions in IDL cholesterol, LDL cholesterol:HDL cholesterol ratio, apoB100, IDL apoB100, LDL apoB100, and the apoB100/apo A-I ratio (Table 2). Isoenergetic replacement of dietary SFAs with UFAs also significantly reduced the cholesterol and apoB100 concentration of the largest less-dense LDL subfraction (LDL-I); and cholesterol, TAG, and apoB100 concentrations in the LDL subfraction of intermediate size and density (LDL-II) (Table 2). There were no significant effects of diet on VLDL and HDL subfractions, other than a significant decrease in the concentration of TAG in the smallest subfraction VLDL-5, in the concentration of apoA2 in the HDL-1 subfraction, and concentrations of apoA1 and apoA2 in the HDL-2 subfraction (Supplementary Table 3).

**TABLE 2:** NMR plasma lipid and lipoprotein subclass analysis in adult males at baseline and after following the higher-SFA/lower-UFA and lower-SFA/higher-UFA diets, each for 4 wk^1^.

NMR plasma lipid and lipoprotein subfractions	Baseline	Higher-SFA/lower-UFA	Lower-SFA/higher-UFA	Difference (Δ)	*P*
NMR lipids					
Total cholesterol, mmol/L	4.81 (4.63, 5.02)	4.95 (4.79, 5.12)	4.57 (4.40, 4.73)	−0.39 (−0.58, −0.20)	<0.0001
VLDL cholesterol, mmol/L	0.40 (0.35, 0.45)	0.39 (0.35, 0.42)	0.38 (0.34, 0.41)	−0.01 (−0.06, 0.03)	0.630
IDL cholesterol, mmol/L	0.23 (0.21, 0.26)	0.24 (0.22, 0.26)	0.19 (0.17, 0.21)	−0.05 (−0.07, −0.02)	<0.0001
LDL cholesterol^2^, mmol/L	2.82 (2.69, 2.97)	2.97 (2.84, 3.09)	2.64 (2.51, 2.76)	−0.33 (−0.47, −0.20)	<0.0001
HDL cholesterol, mmol/L	1.37 (1.32, 1.43)	1.38 (1.34, 1.43)	1.35 (1.30, 1.40)	−0.04 (−0.09, 0.02)	0.192
Total TAG^2^, mmol/L	1.11 (1.01, 1.21)	1.09 (1.01, 1.18)	1.07 (0.99, 1.16)	−0.02 (−0.11, 0.07)	0.300
VLDL-TAG^2^, mmol/L	0.76 (0.68, 0.84)	0.73 (0.66, 0.80)	0.74 (0.66, 0.81)	0.01 (−0.06, 0.08)	0.851
IDL-TAG^2^, mmol/L	0.10 (0.08, 0.11)	0.10 (0.08, 0.11)	0.09 (0.07, 0.10)	−0.01 (−0.02, 0.01)	0.322
LDL-TAG, mmol/L	0.15 (0.15, 0.16)	0.16 (0.15, 0.16)	0.15 (0.14, 0.15)	−0.01 (−0.02, 0)	0.013
HDL-TAG, mmol/L	0.11 (0.10, 0.11)	0.11 (0.10, 0.11)	0.10 (0.10, 0.11)	−0.004 (−0.010, 0.001)	0.119
Total apoB100, g/L	0.76 (0.73, 0.80)	0.79 (0.76, 0.82)	0.73 (0.70, 0.76)	−0.06 (−0.09, −0.03)	<0.0001
VLDL apoB100, g/L	0.07 (0.07, 0.08)	0.07 (0.06, 0.07)	0.07 (0.06, 0.07)	0.001 (−0.004, 0.007)	0.634
IDL apoB100, g/L	0.04 (0.03, 0.04)	0.04 (0.04, 0.04)	0.03 (0.03, 0.03)	−0.01 (−0.01, 0)	<0.0001
LDL apoB100^2^, g/L	0.64 (0.61, 0.67)	0.67 (0.65, 0.70)	0.61 (0.58, 0.63)	−0.06 (−0.09, −0.04)	<0.0001
Total apoA1, g/L	1.37 (1.33, 1.40)	1.38 (1.35, 1.41)	1.34 (1.31, 1.37)	−0.04 (−0.07, 0)	0.042
Total apoA2, g/L	0.32 (0.31, 0.33)	0.32 (0.31, 0.32)	0.31 (0.30, 0.32)	−0.01 (−0.02, 0)	0.107
HDL apoA1, g/L	1.36 (1.32, 1.40)	1.37 (1.34, 1.40)	1.33 (1.30, 1.36)	−0.04 (−0.07, −0.01)	0.020
HDL apoA2, g/L	0.31 (0.31, 0.32)	0.31 (0.30, 0.32)	0.30 (0.30, 0.31)	−0.01 (−0.02, 0)	0.072
LDL cholesterol:HDL cholesterol ratio	2.13 (2.00, 2.26)	2.23 (2.13, 2.32)	2.04 (1.94, 2.13)	−0.19 (−0.29, −0.09)	<0.0001
ApoB100:apoAI ratio	0.57 (0.54, 0.60)	0.58 (0.56, 0.60)	0.55 (0.53, 0.57)	−0.03 (−0.05, −0.01)	0.001
NMR LDL subfractions^2^^,^^3^					
LDL-I cholesterol, mmol/L	0.55 (0.52, 0.58)	0.57 (0.54, 0.59)	0.51 (0.48, 0.53)	−0.06 (−0.09, −0.03)	<0.0001
LDL-II cholesterol, mmol/L	1.80 (1.70, 1.91)	1.93 (1.84, 2.02)	1.65 (1.56, 1.74)	−0.28 (−0.37, −0.19)	<0.0001
LDL-III cholesterol, mmol/L	0.47 (0.43, 0.51)	0.48 (0.45, 0.51)	0.48 (0.45, 0.51)	0 (−0.03, 0.04)	0.637
LDL-I TAG, mmol/L	0.04 (0.04, 0.04)	0.04 (0.04, 0.05)	0.04 (0.04, 0.04)	0	0.605
LDL-II TAG, mmol/L	0.07 (0.07, 0.08)	0.08 (0.07, 0.08)	0.07 (0.06, 0.07)	−0.01 (−0.02, −0.01)	<0.0001
LDL-III TAG, mmol/L	0.04 (0.03, 0.04)	0.04 (0.03, 0.04)	0.04 (0.04, 0.04)	0	0.694
LDL-I apoB100, g/L	0.11 (0.10, 0.11)	0.11 (0.11, 0.11)	0.10 (0.10, 0.11)	−0.01 (−0.01, 0)	0.0017
LDL-II apoB100, g/L	0.40 (0.38, 0.43)	0.43 (0.41, 0.45)	0.37 (0.35, 0.39)	−0.06 (−0.08, −0.04)	<0.0001
LDL-III apoB100, g/L	0.14 (0.13, 0.15)	0.14 (0.13, 0.15)	0.15 (0.14, 0.15)	0 (−0.01, 0.01)	0.942

Abbreviations: Apo, apolipoprotein; IDL, intermediate density lipoprotein; TAG, triacylglycerol; SFA, saturated fatty acid; UFA, unsaturated fatty acid.

1 Values (*n* = 105) for higher-SFA/lower-UFA, lower-SFA/higher-UFA and Δ, are estimated marginal means with 95% confidence intervals derived from a mixed linear model adjusted for age, BMI, baseline value of the measured outcome, and study center. Δ (Delta) denotes difference between the 2 diets and is calculated as lower-SFA/higher-UFA minus higher-SFA/lower-UFA. The *P* value represents the pairwise comparisons of the estimated marginal means between the 2 diets. Baseline (visit 1) indicates values before the dietary intervention and are presented as unadjusted means with 95% confidence intervals. NMR measured outcomes are considered secondary and a more conservative *P* value of <0.01 was considered statistically significant.

2 Indicates data that were log transformed before statistical analysis; these data are presented untransformed and adjusted for age, BMI, baseline value of the measured outcome and study center.

3 Concentration of cholesterol, TAG and apoB100 in LDL subfractions (LDL-I=large size: 27.5–27.0 nm; LDL-II=intermediate size: 27.0–25.5 nm; LDL-III =small size: 25.5–24.2 nm).

### Plasma noncholesterol sterols as markers of intestinal absorption and endogenous synthesis of cholesterol

Isoenergetic replacement of SFAs with UFAs was accompanied by significant increases in all 3 serum noncholesterol sterol biomarkers of cholesterol absorption in the intestine (β-sitosterol, cholestanol, and campesterol (Table 3). Biomarkers of whole-body cholesterol synthesis either significantly increased (desmosterol) or showed no effect (lathosterol) (Table 3).

**TABLE 3:** Markers of intestinal cholesterol absorption and endogenous cholesterol synthesis in adult males at baseline and after following the higher-SFA/lower-UFA and lower-SFA/higher-UFA diets, each for 4 wk^1^.

Outcome	Baseline	Higher-SFA/lower-UFA	Lower-SFA/higher-UFA	Difference (Δ)	*P*
Sitosterol	1.38 (1.29, 1.48)	0.97 (0.94, 1.01)	1.31 (1.27, 1.34)	0.33 (0.30, 0.37)	<0.0001
Cholestanol	1.63 (1.55, 1.70)	1.25 (1.22, 1.28)	1.44 (1.41, 1.47)	0.19 (0.16, 0.22)	<0.0001
Campesterol^2^	1.29 (1.21, 1.37)	0.91 (0.87, 0.94)	1.14 (1.11, 1.18)	0.24 (0.20, 0.27)	<0.0001
Lathosterol	1.32 (1.22, 1.41)	1.13 (1.09, 1.16)	1.08 (1.04, 1.12)	−0.05 (−0.09, 0.00)	0.068
Desmosterol	0.76 (0.73, 0.79)	0.61 (0.59, 0.63)	0.66 (0.65, 0.68)	0.06 (0.04, 0.08)	<0.0001

Abbreviations: TC, total cholesterol; SFA, saturated fatty acid; UFA, unsaturated fatty acid.

1 Values (*n*=108) for higher-SFA/lower-UFA, lower-SFA/higher-UFA and Δ, are estimated marginal means with 95% confidence intervals derived from a mixed linear model adjusted for age, BMI, baseline value of the measured outcome, and study center. Δ (Delta) denotes difference between the 2 diets and is calculated as lower-SFA/higher-UFA minus higher-SFA/lower-UFA. The *P* value represents the pairwise comparisons of the estimated marginal means between the 2 diets. The outcome BMI was adjusted only for age, baseline value, and study center. Baseline (visit 1) indicates values before the dietary intervention and are presented as unadjusted means with 95% confidence intervals. All noncholesterol sterols are presented as a ratio to total cholesterol. *P* < 0.01 was considered statistically significant for all secondary outcomes.

2 Indicates data that were log transformed before statistical analysis; these data are presented untransformed and adjusted for age, BMI, baseline value of the measured outcome and study center.

### Gene expression in circulating PBMCs

The fold change in the PBMC mRNA expression in response to the higher-SFA/lower-UFA and lower-SFA/higher-UFA diets are shown in Figure 1. There was a significant upregulation in the mRNA expression for the *LDL-receptor* (higher-SFA/lower-UFA: 1.04; 95% CI: 0.91, 1.17; lower-SFA/higher-UFA: 1.24; 95% CI: 1.11, 1.37), *NR1H3* (higher-SFA/lower-UFA: 0.96; 95% CI: 0.88, 1.05; lower-SFA/higher-UFA: 1.09; 95% CI: 1.01, 1.17), and *ABCG1* (higher-SFA/lower-UFA: 0.92; 95% CI: 0.79, 1.05; lower-SFA/higher-UFA: 1.25; 95% CI: 1.12, 1.38) genes after the lower-SFA/higher-UFA diet relative to after the higher-SFA/lower-UFA diet. There was no significant effect of the sequential dietary intervention on the mRNA expression of *SREBF1* (higher-SFA/lower-UFA: 0.99; 95% CI: 0.90, 1.08; lower-SFA/higher-UFA: 1.10; 95% CI: 1.01, 1.19) (Figure 1).

### Interindividual variability in serum LDL cholesterol response and predictors of variation

**FIGURE 2:**
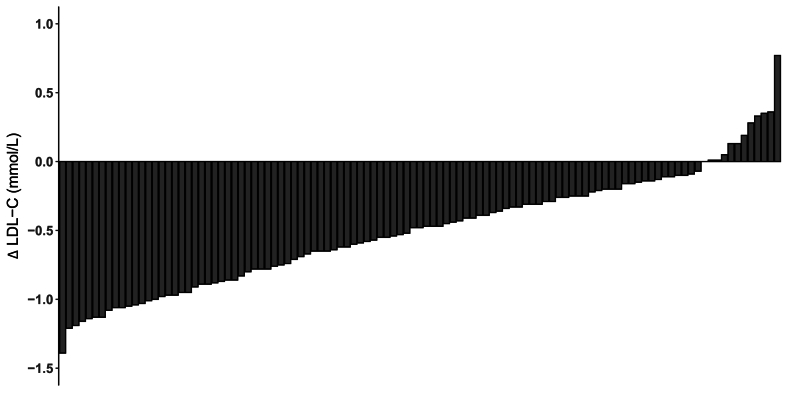
Waterfall plot showing individual changes in serum LDL cholesterol (mmol/L) estimated by Friedewald equation in response to replacing SFA with UFA. Each bar represents an individual participant’s (*n* = 109 adult males) change in LDL cholesterol (Δ LDL-C) after replacing dietary SFA (higher-SFA/lower-UFA diet) with UFA (lower-SFA/higher-UFA diet) for 4 wk.

There was marked interindividual variation in serum LDL cholesterol response to the isoenergetic replacement of SFAs with UFAs, (*n* = 109, range −1.39 to +0.77 mmol/L, mean (SD) −0.5 (0.41) mmol/L) (Figure 2). The serum LDL cholesterol concentration before the lower-SFA/higher-UFA diet (visit 2), explained 27% (adjusted *R*^2^) of the variation in serum LDL cholesterol response upon transitioning from the higher-SFA/lower-UFA to the lower-SFA/higher-UFA diet (*β* coefficient −0.293 mmol/L; 95% CI: −0.410, −0.177; *P* < 0.0001). The reduction in dietary SFA (%TE) between the 2 diets explained 6.7% of the serum LDL cholesterol response (adjusted *R*^2^) (*β* coefficient 0.038 %TE; 95% CI: 0.007, 0.069; *P* = 0.017). When combined, these 2 variables accounted for 33.7% (adjusted *R*^2^) of the difference in serum LDL cholesterol in response to replacing SFA with UFA.

## Discussion

Reduction in SFA intake to below current dietary recommendations of 10% TE, with a matched intake of total fat and other macronutrients, was achieved in a 4-wk, sequential study design, to reproduce the adoption of this dietary advice. This fulfilled the primary aim of the study by lowering serum LDL cholesterol and other CVD risk biomarkers. This dietary exchange produced substantial interindividual variability in the serum LDL cholesterol, one-third of which was explained by the concentration of serum LDL cholesterol before the lower-SFA/higher-UFA diet, combined with the reduction in energy intake from dietary SFA between the 2 diets.

In view of existing evidence for a more favorable effect of n-6 PUFA relative to MUFA in lowering serum LDL cholesterol, the study design included a preference for n-6 PUFA-rich foods in the lower-SFA/higher-UFA diet [12]. The dietary exchange resulted in a marked decrease in our primary outcome of serum LDL cholesterol, as determined indirectly by calculation (Friedewald, −0.50 mmol/L; 95% CI: −0.58, −0.42) and directly in plasma by NMR spectroscopy (−0.33 mmol/L; 95% CI: −0.47, −0.20). The order of magnitude of these responses was consistent with previously published predictive regression and meta-analyses [[24], [25], [26], [27]], and in accordance with reductions in serum LDL cholesterol in response to similar food exchanges in our own, and previous interventions of 4–6 months [[28], [29]]. The lower LDL cholesterol value determined by NMR is consistent with a previous report of lower cholesterol measured in plasma relative to serum [30].

The reduction in plasma LDL cholesterol was predominantly in particles of intermediate density and size (LDL-II), and to a lesser extent, larger LDL (LDL-I). The former, which represented the predominant LDL subfraction in most participants, contains the greatest proportion of cholesterol per LDL particle [31] and has a higher affinity for LDL receptors than both large and small, dense LDL [32]. Moreover, because the concentration of serum LDL cholesterol is regulated, primarily, by its rate of uptake into cells via LDL receptors [33], it follows that the upregulation of the fold change in *LDL-receptor* gene expression, in response to our dietary exchange, results in a reduction in the most receptor-active LDL of intermediate size and density (LDL-II). The LDL cholesterol:apoB ratio has also been used to estimate LDL particle size, with a value of 1.2 and below reflecting a predominance of small-dense LDL and increased CVD risk [34]. In the present study, this ratio decreased from 1.53 to 1.45 after the replacement of SFA with UFA. Although this indicates a reduction in the mean particle size of LDL, ratios above 1.2 were not considered to be of clinical significance with respect to small, dense LDL [34]. The precursor-product relationship between smaller VLDL particles and LDL, as reported in lipoprotein kinetic, trace-labeling studies [35], suggests a link between the decrease in the smallest VLDL-5 subfraction and LDL cholesterol, via a reduction in the production of LDL from this VLDL precursor.

Although there were no significant effects of the diets on blood pressure in the present study, there is evidence that SFA replacement reduces blood pressure [[36], [37]]. We have reported previously a significant decrease in night systolic blood pressure in response to 16 wk of SFAs replacement with UFAs, in participants at increased risk of CVD [29]. In addition to being of longer duration, and in participants at moderate risk of CVD, this previous study also measured 24-h ambulatory blood pressures and focused on vascular function as its primary outcome. Interestingly, this and the present study found significant reductions in serum E-selectin, a cell adhesion molecule involved in the transendothelial migration of leukocytes, a key process in the development of vascular inflammation and atherosclerosis [38]. Increased concentrations of E-selectin have been reported in patients with coronary artery disease [39], and implicated in vascular dysfunction, tissue injury, and vascular diseases [38]. Although there were no significant changes in other cell adhesion molecules, the reduction in E-selectin could reflect a favorable effect of the dietary exchange on vascular health.

The extent of interindividual variation in serum LDL cholesterol in response to the replacement of SFA has been well documented [[6], [7], [8],[40], [41], [42], [43]]. Regulation of serum LDL cholesterol via the transcription of LDL receptors is driven, in part, by a reciprocal relationship between the endogenous synthesis of cholesterol, primarily in the liver, and its absorption in the intestine, which is key to the LDL cholesterol lowering effect of replacing SFAs with UFAs [[44], [45], [46]]. Although it is reasonable to speculate that the observed increase in serum biomarkers (β-sitosterol, campesterol, and cholestanol) of intestinal cholesterol absorption, following the lower-SFA/higher-UFA diet, could be a reciprocal response to decreased cholesterol synthesis (due to diet-induced upregulation of LDL receptor activity), this was not supported by reductions in serum biomarkers of cholesterol synthesis, lathosterol or desmosterol. Dietary fat-induced adaptation in the lipid composition of cellular membranes between individuals has been reported as a possible origin of serum LDL cholesterol variation but was not considered here [47].

*APOE4* carriers showed significantly higher serum LDL cholesterol compared with *APOE2* carriers. This finding is consistent with the impact of these *APOE* variants on serum LDL cholesterol concentration in European populations [48]. However, the *APOE* polymorphism had no significant effect on the serum LDL cholesterol response to the current dietary exchange, possibly because of the short dietary intervention, low serum LDL cholesterol at baseline, or under-representation of *APOE4* carriers and homozygotes in our participants.

The reduction in energy from dietary SFA (transition from the higher-SFA/lower-UFA to the lower-SFA/higher-UFA diet) and serum concentration of LDL cholesterol before the lower-SFA/higher-UFA diet, explained one-third of the variation in serum LDL cholesterol response. These findings may reflect the extent of influence of SFA removal on serum LDL cholesterol and confirm that the concentration of LDL cholesterol is one of several factors that determine the rate of LDL removal from the blood [49]. The mean reductions in serum and plasma LDL cholesterol supports the clinical efficacy of replacing SFA with UFA. At the same time, these average values conceal variation in the LDL cholesterol response that has implications for the management and extent of CVD risk reduction across the range of LDL responses. A decrease in serum LDL cholesterol in an “LDL-responsive” individual of 1 mmol/L translates to a predicted reduction in risk of a cardiac event that is 2-fold greater (24% decrease for myocardial infarction (MI), 22% for MI + stroke) than a relatively non–LDL-responsive individual (reduction in LDL cholesterol 0.5 mmol/L), irrespective of the baseline serum LDL cholesterol [50]. This highlights the importance of targeting dietary advice to responsive individuals to maximize the clinical efficacy of this dietary guideline.

Strengths of the study include its higher-SFA/lower-UFA run-in and sequential diet (lower-SFA/higher-UFA), which were designed to reproduce a transition by replacing SFAs with UFAs, in line with the national dietary guidelines, and used successfully in a previous study (SATgene) [51]. Implementation of the food exchange model was achieved using commercially available foods, with compliance being confirmed by multiple methods [12], making our dietary approach and findings applicable to free-living populations. Furthermore, a per-protocol approach was chosen a priori to allow evaluation of the efficacy of this public health intervention on traditional and novel CVD risk markers. A possible limitation was the retrospective analysis of *APOE* polymorphism, which resulted in genotype groups of unequal sizes. A dietary intervention of 4 wk may also have been insufficient to observe significant differences in secondary outcome measures and may have underestimated the contribution of these outcomes to the variation in LDL cholesterol response. In attempting to control for the confounding effects of hormones on our primary outcome, our study was limited to male participants. Despite a lack of evidence for the sex-specific effects of replacing SFA with UFA on serum LDL cholesterol, this restriction limits the translation of our findings to males only.

In conclusion, this study supports the efficacy of dietary guidelines to reduce SFA to no >10% TE, with replacement with UFAs, in lowering serum LDL cholesterol and other CVD risk factors in only 4 wk. Despite evidence of significant changes in secondary endpoints associated with the LDL-lowering effect of replacing SFAs, only serum LDL cholesterol concentration after the higher-SFA/lower-UFA diet, and reduction in dietary SFA intake predicted variation in the serum LDL cholesterol response. Future studies involving isotopic trace-labeling of dietary fats may provide further insight into the genetic and metabolic origins of this phenomenon.

## Acknowledgments

We thank Dr Nicola Jackson for technical support, Rada Mihaylova and Karen Jenkins for clinical support, and Professor Stine Marie Ulven for providing the assay ID information for the genes measured in the PBMC.

## Author contributions

The authors’ responsibilities were as follows – BAG, JAL, KGJ, BAF, MDR, JS, AK, RA: designed research; AK, RA, EO, LS, GW, KGJ, HA: conducted research; AK, RA, LS: analyzed data or performed statistical analysis; AK, BAG, JAL: wrote the article; BAG, JAL: had primary responsibility for final content; and all authors have contributed to the interpretation of the data, read and approved the final manuscript.

## Conflict of interest

JAL is Deputy Chair of the UK Government's Scientific Advisory committee on Nutrition (SACN). JAL (Chair), LS and KGJ were members of the International Life Science Institute (ILSI) Europe expert group on “Update on health effects of different dietary saturated fats” (2017–2022). The other authors have no conflicts of interest or competing interests to declare.

## Funding

The RISSCI study was funded by the Biotechnology and Biological Sciences Research Council (BBSRC) project “Mechanisms to Explain Variation in Serum Low Density Lipoprotein Cholesterol Response to Dietary Saturated Fat” (Project references: BB/P010245/1 and BB/P009891/1).

## Data availability

Data described in the manuscript will be made available upon request to the corresponding author pending application and approval.
