# Supplementary material for: Variation of LDL cholesterol in response to the replacement of saturated with unsaturated fatty acids: a nonrandomized, sequential dietary intervention; the Reading, Imperial, Surrey, Saturated fat Cholesterol Intervention (“RISSCI”-1) study
Source: Am J Clin Nutr. 2024 Aug 5;120(4):854–63. doi: 10.1016/j.ajcnut.2024.07.032 (PMC11473524; doi:10.1016/j.ajcnut.2024.07.032)
Supplement: Multimedia component 1 [file mmc1.pdf]

Online Supplementary Material

Title: Variation of LDL-cholesterol in response to the replacement of saturated with unsaturated fatty acids: a non-randomised, sequential dietary intervention study (RISSCI-1), by Koutsos et al. (2024)

SUPPLEMENTARY MATERIALS AND METHODS

For the stepwise regression analysis (n=52) to identify predictors of LDL-C change after transitioning from a Higher-SFA/Lower-UFA to a Lower-SFA/Higher-UFA diet, the following variables were selected:

Response/dependent variable: The difference (delta) in LDL-C (mmol/L) between visit 3 (lower-SFA/higher-UFA) and visit 2 (higher-SFA/lower-UFA).

Predictor variables:

- Participant Age at baseline (visit 1).
- Genotype carrier code (categorical variable: i.E3/E3 carriers; ii.E2 carriers including E2/E2 and E2/E3 carriers; iii. E4 carriers including E4/E4 and E3/E4 carriers).
- Dietary intake data, presented as the difference between visit 3 (lower-SFA/higher-UFA) and visit 2 (higher-SFA/lower-UFA) for the following variables:

Energy (kcal), fibre (g), cholesterol (mg), SFA (% of TE), MUFA (% of TE), PUFA (% of TE), omega-3 FAs (% of TE), omega-6 FAs (% of TE), trans FAs (% of TE), protein (% of TE), carbohydrates (% of TE); UFA/SFA ratio and net increase in UFA (g) between the two diets.

- Other variables all measured at visit 2 (higher-SFA/lower-UFA):

BMI (kg/m^2), waist circumference (cm), glucose (mmol/L), insulin (pmol/L), TC (mmol/L), HDL-C (mmol/L), TAG (mmol/L), LDL-C (mmol/L), remnant lipoprotein cholesterol (mmol/L), apoB (g/L), E-selectin (ng/mL), P-Selectin (ng/mL), sitosterol, cholestanol, campesterol, desmosterol ratios; NMR LDL subfractions: LDL-I cholesterol (mmol/L), LDL-II cholesterol (mmol/L), LDL-III cholesterol (mmol/L), LDL-I apoB100 (g/L), LDL-II apoB100 (g/L), LDL-III apoB100 (g/L).

SUPPLEMENTARY TABLES

Supplementary Table 1. Anthropometric measurements, blood pressure and blood biochemical analysis according to *APOE* carrier group, in adult males at baseline and after following the higher-SFA/lower-UFA and lower-SFA/higher-UFA diets, each for 4 weeks[1].

	***E2* carriers (n=15)**				***E3/E3* (n=70)**				***E4* carriers (n=21)**				***P***		
	**Baseline**	**Higher-SFA/ Lower-UFA**	**Lower-SFA/ Higher-UFA**	**Δ**	**Baseline**	**Higher-SFA/ Lower-UFA**	**Lower-SFA/ Higher-UFA**	**Δ**	**Baseline**	**Higher-SFA/ Lower-UFA**	**Lower-SFA/ Higher-UFA**	**Δ**	**D**	**G**	**DxG**
Anthropometrics															
Weight, kg	78.1 (74.9, 81.4)	79.6 (78.9, 80.3)	79.6 (78.9, 80.3)	0.01 (-0.68, 0.71)	80.8 (78.7, 82.8)	79.6 (79.3, 79.9)	79.4 (79.1, 79.7)	-0.17 (-0.49, 0.15)	77.2 (74.1, 80.3)	79.6 (79.0, 80.1)	79.3 (78.8, 79.9)	-0.22 (-0.81, 0.36)	0.25	0.95	0.73
BMI, kg/m^2	24.4 (23.3, 25.6)	25.2 (24.9, 25.4)	25.2 (25.0, 25.4)	0.01 (-0.21, 0.23)	25.4 (24.9, 26.0)	25.2 (25.1, 25.3)	25.1 (25.0, 25.2)	-0.04 (-0.14, 0.06)	24.7 (23.7, 25.7)	25.1 (24.9, 25.3)	25.0 (24.9, 25.2)	-0.09 (-0.27, 0.10)	0.30	0.85	0.61
Waist, cm	89.3 (86.4, 92.3)	91.9 (90.6, 93.2)	92.0 (90.7, 93.4)	0.12 (-1.77, 2.01)	93.1 (91.6, 94.7)	91.6 (90.9, 92.2)	91.2 (90.5, 91.8)	-0.40 (-1.30, 0.49)	91.4 (88.5, 94.2)	91.9 (90.8, 93.0)	92.3 (91.2, 93.4)	0.39 (-1.24, 2.02)	0.91	0.33	0.42
Hip, cm	102 (100, 103)	102 (101, 103)	102 (101, 103)	0.13 (-1.61, 1.86)	103 (102, 104)	102 (101, 102)	101 (101, 102)	-0.41 (-1.21, 0.38)	101 (99, 103)	103 (102, 103)	103 (102, 104)	0.83 (-0.61, 2.27)	0.51	0.04	0.09
Waist:hip ratio	0.88 (0.85, 0.90)	0.90 (0.89, 0.91)	0.90 (0.89, 0.91)	0.00 (-0.02, 0.02)	0.91 (0.90, 0.92)	0.90 (0.89, 0.90)	0.90 (0.89, 0.90)	0.00 (-0.01, 0.01)	0.90 (0.89, 0.92)	0.90 (0.88, 0.91)	0.89 (0.88, 0.90)	0.00 (-0.02, 0.01)	0.67	0.76	0.89
Fat, %	20.2 (18.1, 22.3)	21.9 (21.2, 22.7)	21.6 (20.9, 22.4)	-0.32 (-1.25, 0.61)	21.7 (20.7, 22.7)	21.4 (21.0, 21.7)	21.2 (20.8, 21.5)	-0.20 (-0.63, 0.24)	21.5 (19.4, 23.7)	21.2 (20.5, 21.8)	20.8 (20.2, 21.5)	-0.36 (-1.14, 0.43)	0.05	0.24	0.85
Blood pressure (BP), mmHg															
Systolic BP	120 (116, 123)	123 (120, 127)	124 (120, 127)	0.57 (-4.48, 5.62)	121 (119, 123)	119 (117, 121)	120 (118, 122)	1.17 (-1.28, 3.62)	118 (114, 122)	120 (117, 123)	118 (115, 121)	-2.47 (-6.81, 1.86)	0.76	0.06	0.11
Diastolic BP	78.5 (75.7, 81.3)	79.6 (76.6, 82.6)	81.7 (78.7, 84.6)	2.07 (-2.77, 6.91)	77.8 (76.5, 79.2)	76.1 (74.6, 77.5)	76.2 (74.8, 77.7)	0.17 (-2.17, 2.51)	76.7 (74.0, 79.4)	78.2 (75.7, 80.8)	76.3 (73.7, 78.9)	-1.95 (-6.10, 2.21)	0.90	0.01	0.18
PP	41.7 (37.6, 45.8)	43.4 (40.1, 46.8)	41.9 (38.5, 45.2)	-1.57 (-7.42, 4.28)	43.0 (40.8, 45.3)	43.1 (41.5, 44.7)	44.1 (42.5, 45.8)	1.02 (-1.81, 3.84)	40.9 (36.5, 45.2)	42.2 (39.4, 45.1)	41.5 (38.6, 44.4)	-0.74 (-5.76, 4.28)	0.65	0.40	0.42
Blood biochemical analysis															
TC, mmol/L	4.62 (4.33, 4.92)	5.36 (5.07, 5.65)	4.89 (4.60, 5.18)	-0.47 (-0.86, -0.08)	5.16 (5.00, 5.32)	5.47 (5.34, 5.61)	4.79 (4.66, 4.92)	-0.68 (-0.86, -0.50)	5.58 (5.22, 5.95)	5.35 (5.10, 5.59)	4.66 (4.42, 4.91)	-0.68 (-1.01, -0.35)	<0.01	0.60	0.36
LDL-C, mmol/L [3]	2.67 [a] (2.41, 2.93)	3.28 (3.04, 3.52)	2.88 (2.64, 3.12)	-0.40 (-0.71, -0.09)	3.21 [ab] (3.07, 3.34)	3.43 (3.32, 3.54)	2.90 (2.79, 3.01)	-0.53 (-0.68, -0.39)	3.57 [b] (3.25, 3.89)	3.33 (3.13, 3.53)	2.86 (2.65, 3.06)	-0.48 (-0.74, -0.22)	<0.01	0.67	0.52
HDL-C, mmol/L	1.50 (1.37, 1.63)	1.51 (1.42, 1.61)	1.42 (1.32, 1.52)	-0.09 (-0.23, 0.04)	1.39 (1.34, 1.45)	1.49 (1.44, 1.53)	1.38 (1.33, 1.42)	-0.11 (-0.17, -0.05)	1.45 (1.36, 1.55)	1.46 (1.38, 1.54)	1.33 (1.25, 1.41)	-0.13 (-0.24, -0.01)	<0.01	0.41	0.86
TAG, mmol/L	1.10 (0.74, 1.46)	1.27 (1.09, 1.46)	1.33 (1.14, 1.52)	0.06 (-0.22, 0.34)	1.25 (1.12, 1.38)	1.22 (1.14, 1.31)	1.14 (1.05, 1.23)	-0.09 (-0.21, 0.04)	1.20 (0.87, 1.52)	1.19 (1.03, 1.35)	1.02 (0.86, 1.17)	-0.17 (-0.41, 0.06)	0.13	0.18	0.19
NEFAs, mmol/L [3]	0.37 [a] (0.28, 0.46)	0.44 (0.36, 0.52)	0.42 (0.34, 0.50)	-0.02 (-0.17, 0.13)	0.43 [ab] (0.40, 0.45)	0.40 (0.36, 0.44)	0.43 (0.39, 0.47)	0.04 (-0.03, 0.11)	0.55 [b] (0.48, 0.63)	0.41 (0.34, 0.48)	0.43 (0.36, 0.50)	0.02 (-0.11, 0.14)	0.66	0.88	0.62
TC:HDL-C ratio	3.17 (2.93, 3.42)	3.79 (3.57, 4.02)	3.64 (3.41, 3.87)	-0.15 (-0.44, 0.14)	3.87 (3.70, 4.03)	3.87 (3.76, 3.97)	3.62 (3.52, 3.73)	-0.25 (-0.38, -0.11)	4.06 (3.62, 4.49)	3.81 (3.62, 4.01)	3.63 (3.43, 3.82)	-0.19 (-0.43, 0.06)	<0.01	0.95	0.62

LDL-C:HDL-C ratio	1.85 (1.63, 2.08)	2.35 (2.16, 2.54)	2.18 (1.99, 2.37)	-0.17 (-0.43, 0.10)	2.43 (2.29, 2.57)	2.45 (2.36, 2.54)	2.22 (2.13, 2.30)	-0.24 (-0.36, -0.12)	2.63 (2.26, 3.00)	2.38 (2.22, 2.55)	2.25 (2.09, 2.41)	-0.13 (-0.36, 0.09)	<0.01	0.76	0.44
Non HDL-C, mmol/L [3]	3.13 **a** (2.84, 3.41)	3.86 (3.60, 4.12)	3.48 (3.22, 3.74)	-0.38 (-0.71, -0.05)	3.78 **ab** (3.62, 3.94)	3.99 (3.87, 4.11)	3.41 (3.29, 3.53)	-0.57 (-0.73, -0.42)	4.13 **b** (3.77, 4.50)	3.89 (3.67, 4.11)	3.34 (3.12, 3.56)	-0.55 (-0.83, -0.27)	<0.01	0.73	0.30
Remnant lipoprotein cholesterol, mmol/L	0.46 (0.40, 0.52)	0.57 (0.48, 0.66)	0.61 (0.52, 0.70)	0.04 (-0.09, 0.17)	0.56 (0.52, 0.61)	0.56 (0.52, 0.61)	0.52 (0.48, 0.57)	-0.04 (-0.10, 0.02)	0.57 (0.48, 0.65)	0.54 (0.47, 0.62)	0.47 (0.40, 0.55)	-0.07 (-0.18, 0.04)	0.26	0.27	0.17
ApoB, g/L	0.72 (0.63, 0.81)	0.83 (0.78, 0.88)	0.77 (0.72, 0.82)	-0.06 (-0.14, 0.02)	0.87 (0.83, 0.91)	0.87 (0.84, 0.89)	0.77 (0.74, 0.79)	-0.10 (-0.14, -0.06)	0.90 (0.79, 1.01)	0.84 (0.80, 0.89)	0.75 (0.71, 0.80)	-0.09 (-0.16, -0.02)	<0.01	0.55	0.39
Glucose, mmol/L	5.30 (5.10, 5.51)	5.21 (5.04, 5.38)	5.28 (5.11, 5.45)	0.07 (-0.19, 0.33)	5.23 (5.16, 5.30)	5.21 (5.13, 5.29)	5.24 (5.17, 5.32)	0.03 (-0.08, 0.15)	5.17 (5.04, 5.31)	5.22 (5.08, 5.36)	5.22 (5.08, 5.37)	0.00 (-0.22, 0.22)	0.40	0.97	0.84
Insulin, pmol/L [2]	24.3 (20.1, 28.5)	34.8 (27.9, 41.6)	34.6 (27.8, 41.5)	0.15 (-9.86, 10.15)	28.4 (25.9, 30.8)	35.3 (32.1, 38.6)	34.1 (30.9, 37.4)	1.21 (-3.49, 5.91)	50.4 (19.2, 81.6)	33.2 (27.3, 39.0)	28.7 (22.9, 34.6)	4.47 (-3.99, 12.93)	0.37	0.28	0.13
CRP, mg/l [2]	0.95 (0.48, 1.43)	1.35 (0.88, 1.82)	1.14 (0.67, 1.61)	-0.20 (-0.84, 0.43)	1.49 (1.23, 1.75)	1.29 (1.07, 1.50)	1.15 (0.93, 1.37)	-0.14 (-0.43, 0.16)	0.55[§] (0.39, 0.71)	0.98 (0.58, 1.37)	1.04 (0.65, 1.43)	0.06 (-0.46, 0.58)	0.09	0.17	0.91
VCAM-1, ng/mL	467 (429, 504)	469 (418, 520)	456 (405, 507)	-13.0 (-87.9, 61.8)	476 (445, 506)	467 (444, 490)	470 (447, 493)	3.0 (-30.7, 36.7)	472 (428, 517)	478 (436, 519)	505 (463, 547)	27.3 (-33.8, 88.4)	0.63	0.50	0.44
ICAM-1, ng/mL	203 (185, 221)	235 (215, 255)	216 (196, 236)	-19.5 (-51.9, 13.0)	224 (212, 236)	233 (224, 242)	229 (220, 238)	-4.6 (-19.2, 10.0)	275 (237, 314)	228 (211, 245)	224 (207, 241)	-3.7 (-30.2, 22.9)	0.07	0.72	0.46
E-selectin, ng/mL	24.9 (22.0, 27.9)	26.6 (24.4, 28.8)	25.5 (23.3, 27.7)	-1.14 (-4.28, 2.00)	25.8 (24.1, 27.5)	26.3 (25.3, 27.3)	25.0 (24.0, 25.9)	-1.33 (-2.74, 0.08)	28.3 (25.1, 31.5)	25.7 (23.9, 27.5)	24.1 (22.3, 25.9)	-1.59 (-4.15, 0.98)	0.01	0.61	0.95
P-selectin, ng/mL	27.2 (24.8, 29.6)	28.3 (26.0, 30.6)	26.7 (24.4, 29.0)	-1.61 (-5.07, 1.85)	26.4 (25.1, 27.7)	27.1 (26.0, 28.1)	26.3 (25.2, 27.3)	-0.77 (-2.33, 0.79)	27.9 (25.7, 30.1)	28.0 (26.1, 29.9)	26.4 (24.5, 28.2)	-1.63 (-4.45, 1.20)	0.02	0.68	0.66
PCSK9, ng/mL	188 (172, 204)	200 (182, 218)	199 (181, 216)	-1.70 (29.81, 26.41)	194 (187, 201)	194 (186, 202)	200 (192, 208)	6.05 (-6.61, 18.71)	184 (167, 201)	191 (176, 206)	184 (169, 198)	-7.64 (30.60, 15.31)	0.80	0.36	0.29

[1] Values for Higher-SFA/Lower-UFA, Lower-SFA/Higher-UFA and Δ, are estimated marginal means with 95% confidence intervals derived from a mixed linear model adjusted for age, BMI, baseline value of the measured outcome, and study centre. Δ (delta) denotes difference between the two diets and is calculated as Lower-SFA/Higher-UFA minus Higher-SFA/Lower-UFA. The outcome BMI was adjusted only for age, baseline value and study centre. Baseline (visit 1) indicates values before the dietary intervention and are presented as unadjusted means with 95% confidence intervals. For all variables, n=106: *E3/E3* carriers (n=70); *E2* carriers (n=15) were combined to include *E2/E2* and *E2/E3* carriers; *E4* carriers (n=21) were combined to include *E4/E4* and *E3/E4* carriers. For changes in blood cholesterol (primary outcome) TC, LDL-C and HDL-C, $P < 0.05$ was considered statistically significant. All other measured outcomes are considered secondary and a more conservative *P* value of 0.01 was considered statistically significant.

[2] Indicates data that were log transformed prior to statistical analysis; these data are presented untransformed and adjusted for age, BMI, baseline value of the measured outcome and study centre.

[3] ANOVA was used to evaluate the influence of carrier codes on baseline (visit 1) blood pressure and other CVD risk markers; $P<0.01$ was considered statistically significant, a, b superscript letters were used for the post hoc analysis. The same superscript letter indicates no significant difference, while different superscript letters denote significant differences ($P<0.01$).

Abbreviations: Apo, apolipoprotein; BP, blood pressure; CRP, C-reactive protein; D, Diet; D x G, diet genotype interaction; G, genotype; HDL-C, high density lipoprotein cholesterol; ICAM-1, intercellular cell adhesion molecule 1; LDL-C, low density lipoprotein cholesterol; n, refers to the number of participants for each measured outcome; NEFA, non-esterified fatty acid; PCSK9, protein convertase subtilisin/kexin type 9; PP, pulse pressure; SFA, saturated fatty acids; TAG, triacylglycerol; TC, total cholesterol; UFA, unsaturated fatty acids; VCAM-1, vascular cell adhesion molecule 1.

Supplementary Table 2. Markers of intestinal cholesterol absorption and endogenous cholesterol synthesis according to *APOE* carrier group in adult males at baseline and after following the higher-SFA/lower-UFA and lower-SFA/higher-UFA diets, each for 4 weeks[1].

	***E2* carriers (n=15)**				***E3/E3* (n=70)**				***E4* carriers (n=21)**				***P***		
	**Baseline**	**Higher-SFA/ Lower-UFA**	**Lower-SFA/ Higher-UFA**	**Δ**	**Baseline**	**Higher-SFA/ Lower-UFA**	**Lower-SFA/ Higher-UFA**	**Δ**	**Baseline**	**Higher-SFA/ Lower-UFA**	**Lower-SFA/ Higher-UFA**	**Δ**	**D**	**G**	**D*G**
Sitosterol	1.38 (1.19, 1.57)	1.02 (0.92, 1.12)	1.35 (1.25, 1.45)	0.33 (0.18, 0.48)	1.36 (1.28, 1.44)	0.98 (0.93, 1.02)	1.31 (1.26, 1.35)	0.33 (0.26, 0.40)	1.50 (1.32, 1.67)	0.94 (0.86, 1.03)	1.30 (1.22, 1.39)	0.36 (0.23, 0.49)	<0.001	0.55	0.83
Cholestanol	1.61 (1.45, 1.78)	1.32 (1.23, 1.41)	1.47 (1.38, 1.55)	0.15 (0.02, 0.27)	1.57 (1.51, 1.63)	1.25 (1.20, 1.29)	1.43 (1.39, 1.47)	0.19 (0.13, 0.24)	1.83 (1.69, 1.97)	1.24 (1.17, 1.32)	1.44 (1.37, 1.52)	0.20 (0.09, 0.31)	<0.001	0.45	0.64
Campesterol	1.31 (1.08, 1.54)	0.99 (0.90, 1.09)	1.23 (1.13, 1.32)	0.23 (0.11, 0.36)	1.27 (1.21, 1.34)	0.90 (0.86, 0.95)	1.13 (1.08, 1.17)	0.22 (0.16, 0.28)	1.34 (1.22, 1.45)	0.90 (0.82, 0.98)	1.15 (1.07, 1.23)	0.25 (0.14, 0.36)	<0.001	0.13	0.82
Lathosterol	1.17 (1.03, 1.30)	1.05 (0.95, 1.16)	1.15 (1.04, 1.25)	0.09 (-0.09, 0.28)	1.36 (1.28, 1.45)	1.14 (1.09, 1.19)	1.08 (1.03, 1.13)	-0.05 (-0.14, 0.03)	1.30 (1.13, 1.46)	1.15 (1.06, 1.24)	1.03 (0.94, 1.12)	-0.12 (-0.28, 0.04)	0.36	0.90	0.04
Desmosterol	0.71 (0.66, 0.76)	0.60 (0.55, 0.65)	0.69 (0.65, 0.74)	0.09 (0.02, 0.17)	0.78 (0.75, 0.80)	0.61 (0.59, 0.63)	0.67 (0.64, 0.69)	0.06 (0.02, 0.09)	0.78 (0.73, 0.82)	0.60 (0.55, 0.64)	0.63 (0.59, 0.67)	0.04 (-0.03, 0.10)	<0.001	0.41	0.26

[1]Values for Higher-SFA/Lower-UFA, Lower-SFA/Higher-UFA and Δ, are estimated marginal means with 95% confidence intervals derived from a mixed linear model adjusted for age, BMI, baseline value of the measured outcome, and study centre. Δ (delta) denotes difference between the two diets and is calculated as Lower-SFA/Higher-UFA minus Higher-SFA/Lower-UFA. Baseline (visit 1) indicates values before the dietary intervention and are presented as unadjusted means with 95% confidence intervals. For all variables, n=106: *E3/E3* carriers (n=70); *E2* carriers (n=15) were combined to include *E2/E2* and *E2/E3* carriers; *E4* carriers (n=21) were combined to include *E4/E4* and *E3/E4* carriers. All non-cholesterol sterols are presented as a ratio to total cholesterol. $P < 0.01$ was considered statistically significant for all secondary outcomes.

[2]Indicates data that were log transformed prior to statistical analysis; these data are presented untransformed and adjusted for age, BMI, baseline value of the measured outcome and study centre.

Abbreviations: Apo, apolipoprotein; D, Diet; D x G, diet genotype interaction; G, genotype; SFA, saturated fatty acids; UFA, unsaturated fatty acids.

Supplementary Table 3. NMR lipoprotein subclass analysis in adult males at baseline and after following the higher-SFA/lower-UFA and lower-SFA/higher-UFA diets, each for 4 weeks[1].

Lipoprotein Subclass	Baseline	Higher-SFA/ Lower-UFA	Lower-SFA- Higher-UFA	Difference (Δ)	*P*
VLDL-1					
TAG, mmol/L	0.40 (0.35, 0.45)	0.39 (0.35, 0.44)	0.39 (0.35, 0.44)	-0.003 (-0.047, 0.041)	0.887
Cholesterol[2], mmol/L	0.12 (0.10, 0.14)	0.12 (0.10, 0.13)	0.12 (0.10, 0.13)	0 (-0.017, 0.017)	0.991
VLDL-2					
TAG, mmol/L	0.14 (0.12, 0.16)	0.13 (0.12, 0.15)	0.14 (0.13, 0.16)	0.01 (-0.01, 0.03)	0.277
Cholesterol, mmol/L	0.05 (0.04, 0.06)	0.05 (0.04, 0.05)	0.05 (0.04, 0.06)	0.004 (-0.005, 0.013)	0.381
VLDL-3					
TAG, mmol/L	0.12 (0.10, 0.13)	0.11 (0.10, 0.12)	0.12 (0.10, 0.13)	0.01 (-0.01, 0.02)	0.262
Cholesterol, mmol/L	0.08 (0.07, 0.09)	0.07 (0.06, 0.08)	0.07 (0.07, 0.08)	0.005 (-0.006, 0.015)	0.358
VLDL-4					
TAG, mmol/L	0.09 (0.08, 0.10)	0.09 (0.08, 0.09)	0.08 (0.08, 0.09)	-0.001 (-0.007, 0.004)	0.628
Cholesterol, mmol/L	0.10 (0.09, 0.12)	0.10 (0.09, 0.11)	0.09 (0.08, 0.10)	-0.01 (-0.02, 0)	0.115
VLDL-5					
TAG[2], mmol/L	0.03 (0.02, 0.03)	0.03 (0.03, 0.03)	0.02 (0.02, 0.03)	-0.002 (-0.004, -0.001)	0.003
Cholesterol, mmol/L	0.03 (0.03, 0.03)	0.03 (0.03, 0.03)	0.03 (0.03, 0.03)	-0.001 (-0.005, 0.002)	0.471
HDL-1					
TAG, mmol/L	0.03 (0.03, 0.03)	0.03 (0.03, 0.03)	0.03 (0.03, 0.03)	-0.002 (-0.004, 0)	0.072
Cholesterol[2], mmol/L	0.40 (0.37, 0.43)	0.41 (0.38, 0.44)	0.38 (0.35, 0.42)	-0.02 (-0.06, 0.01)	0.195
ApoA1[2], g/L	0.20 (0.17, 0.22)	0.20 (0.18, 0.22)	0.18 (0.16, 0.20)	-0.02 (-0.04, 0)	0.075
ApoA2[2], g/L	0.02 (0.02, 0.02)	0.02 (0.02, 0.02)	0.02 (0.01, 0.02)	-0.004 (-0.006, -0.002)	<0.0001
HDL-2					
TAG, mmol/L	0.02 (0.02, 0.02)	0.02 (0.02, 0.02)	0.02 (0.02, 0.02)	0 (-0.001, 0)	0.280
Cholesterol[2], mmol/L	0.20 (0.18, 0.21)	0.19 (0.18, 0.21)	0.19 (0.18, 0.20)	-0.01 (-0.02, 0.01)	0.174
ApoA1[2], g/L	0.16 (0.15, 0.17)	0.16 (0.16, 0.17)	0.15 (0.15, 0.16)	-0.01 (-0.02, -0.01)	<0.0001
ApoA2, g/L	0.03 (0.03, 0.03)	0.03 (0.03, 0.03)	0.02 (0.02, 0.03)	-0.003 (-0.005, -0.002)	<0.0001
HDL-3					
TAG, mmol/L	0.02 (0.02, 0.02)	0.02 (0.02, 0.02)	0.02 (0.02, 0.02)	0 (-0.001, 0.001)	0.799
cholesterol, mmol/L	0.24 (0.23, 0.25)	0.24 (0.23, 0.25)	0.24 (0.23, 0.25)	-0.003 (-0.012, 0.006)	0.504
ApoA1, g/L	0.23 (0.23, 0.24)	0.24 (0.23, 0.24)	0.23 (0.22, 0.24)	-0.01 (-0.01, 0)	0.022
ApoA2, g/L	0.06 (0.06, 0.06)	0.06 (0.06, 0.06)	0.06 (0.05, 0.06)	-0.003 (-0.005, 0)	0.029
HDL-4					
TAG[2], mmol/L	0.04 (0.04, 0.04)	0.04 (0.04, 0.04)	0.04 (0.04, 0.04)	-0.001 (-0.003, 0.002)	0.547
cholesterol, mmol/L	0.54 (0.52, 0.56)	0.55 (0.53, 0.56)	0.54 (0.53, 0.56)	-0.004 (-0.019, 0.011)	0.620
ApoA1, g/L	0.76 (0.74, 0.78)	0.77 (0.74, 0.79)	0.76 (0.74, 0.78)	-0.01 (-0.03, 0.01)	0.396
ApoA2[2], g/L	0.20 (0.20, 0.21)	0.20 (0.20, 0.21)	0.20 (0.19, 0.21)	-0.003 (-0.010, 0.004)	0.665

[1] Values (n=105) for Higher-SFA/Lower-UFA, Lower-SFA/Higher-UFA and Δ, are estimated marginal means with 95% confidence intervals derived from a mixed linear model adjusted for age, BMI, baseline value of the measured outcome, and study centre. Δ (delta) denotes difference between the two diets and is calculated as Lower-SFA/Higher-UFA minus Higher-SFA/Lower-UFA. The *P*-value represents the pairwise comparisons of the estimated marginal means between the two diets. Baseline (visit 1) indicates values before the dietary intervention and are presented as unadjusted means with 95% confidence intervals. NMR measured outcomes are considered secondary and a more conservative *P* value of 0.01 was considered statistically significant. Densities of lipoprotein subfractions, HDL-1: 1.063-1.100 kg/L, HDL-2: 1.100-1.112 kg/L, HDL-3: 1.112-1.125 kg/L, HDL-4: 1.125-1.210 kg/L. The density ranges for VLDL subfractions 1-5 are specified in *Lindgren FT, Jensen LL, Hatch FT (1972) The isolation and quantitative analysis of serum lipoproteins. In: Nelson GJ (ed.) Blood lipids and lipoproteins: Quantitation, composition and metabolism. Wiley-Interscience, New York, p 181-274*

[2]Indicates data that were log transformed prior to statistical analysis; these data are presented untransformed and adjusted for age, BMI, baseline value of the measured outcome and study centre.

Abbreviations: Apo, apolipoprotein; HDL-C, high density lipoprotein cholesterol; LDL-C, low density lipoprotein cholesterol; SFA, saturated fatty acids; TAG, triacylglycerol; UFA, unsaturated fatty acids; VLDL-C, very low density lipoprotein cholesterol.

SUPPLEMENTARY FIGURE

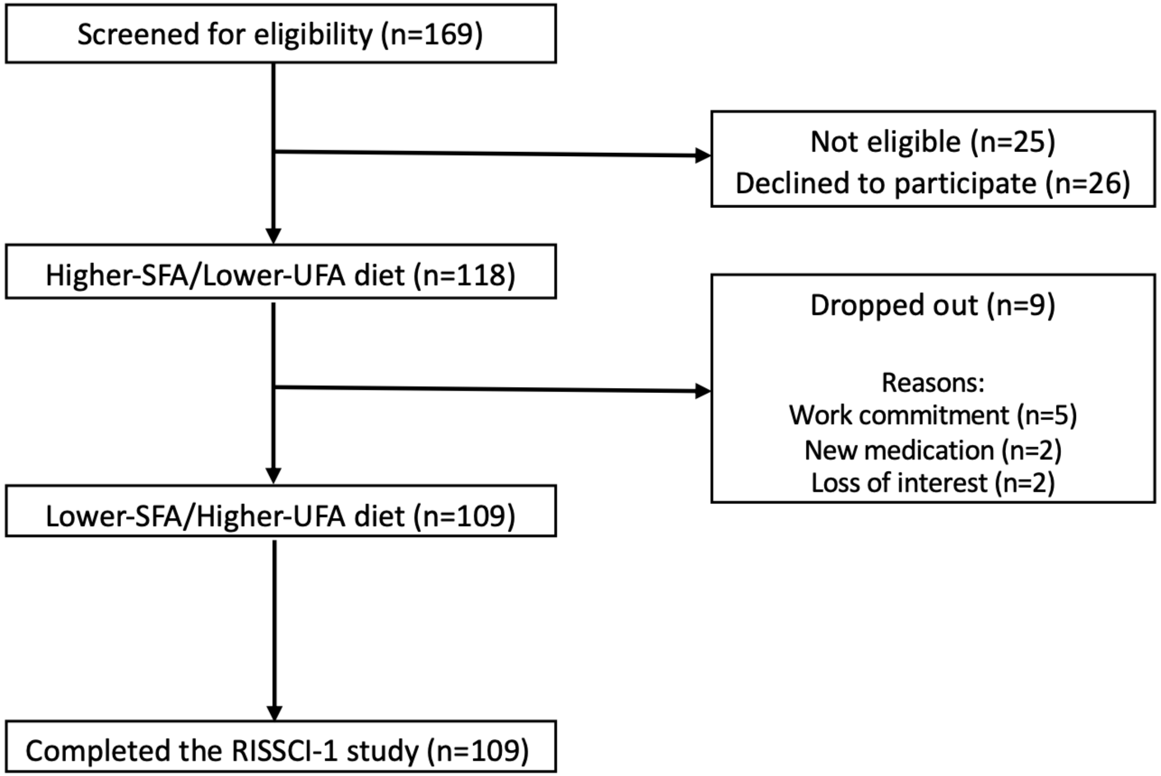

Supplementary Figure 1. Participant flow chart of the Reading, Imperial, Surrey, Saturated fat Cholesterol Intervention (RISSCI-1) study, adapted from Sellem et al. (2022) (12). Abbreviations: SFA, saturated fatty acids; UFA, unsaturated fatty acids.
